# The Effect of Brand Personality on Electronic Word-of-Mouth: Mediation of Brand Love and Moderated Mediation of Brand Experience Sharing

**DOI:** 10.3389/fpsyg.2022.936033

**Published:** 2022-06-29

**Authors:** Manlin Liu, Jinzhe Yan

**Affiliations:** ^1^Department of Business Administration, Gachon University, Seongnam-si, South Korea; ^2^Department of Marketing, College of Business, Gachon University, Seongnam-si, South Korea

**Keywords:** brand personality, brand love, electronic word-of-mouth, brand experience sharing, knowledge payment

## Abstract

The knowledge payment industry will rapidly attract many enterprises that provide knowledge services. This study investigates the interrelationship between brand personality, brand love, and electronic word-of-mouth in the context of knowledge payment. Moreover, this study explored the brand experience sharing boundary condition by adopting a survey. Firstly, the main research results show that brand personality has a significant positive impact on brand love. Secondly, brand love also has a significant positive influence on electronic word-of-mouth. Thirdly, brand experience sharing plays a positive role in regulating brand love and electronic word-of-mouth. This research promotes e-marketing by focusing on brand personality, brand love, e-word of mouth, and other perspectives to improve business operations, user experience, and engagement, providing dedicated products or services to the customer base for profit. As an emerging market, knowledge payment will attract the participation of many knowledge service enterprises.

## Introduction

Cultivating brand loyalty effectively reduces the costs of expanding a corporation’s consumer base. A conclusion drawn from research in western academia in the 1960s shows that the cost of attracting new consumers is five times higher than retaining old customers (Arnaldo et al., 2019). The word “brand” first appeared in the 1870s, when people had little knowledge of the brand. Moreover, the specific concept of the brand made its debut in the modern advertising industry (Li et al., 2012). However, most brand research focuses on how brand factors contribute to conventional brands instead of internet brands.

As the income increases, the consumption demand varies. Especially after the rise of the internet and mobile payment, the need for paid content has increased since 2016, and knowledge payment is derived from it. Knowledge is commercialized and service-oriented under knowledge payment, which is a mode of business and trade. More specifically, knowledge payment means absorbing knowledge and service from the media and internet aside from traditional books and education. Using the new third-party payments allows knowledge to be commercialized and monetized (Wang et al., 2018).

Based on SOR Model (Mehrabian and Russell, 1974), brand personality, brand love, and other theories, the author delves into how electronic word-of-mouth addresses the deficiency in knowledge payment research, offers references on the electronic word-of-mouth of knowledge payment, and expands the application of relevant theories. This research starts from the brand itself and digs deeper into marketing and communication, enriches the references for further studies, and provides the instrument for the future development of knowledge payment.

Based on the analysis of the theoretical background, this study believes that as the Blue Ocean Market, the knowledge payment industry will rapidly attract an enormous number of enterprises that provide knowledge services. These enterprises may resort to some regular marketing methods, including brand marketing. In this research, we attempt to answer the following research questions.

1. What is the relationship between brand personality and brand love?
2. What is the influence of brand love on eWOM?
3. What is the function of consumers with different characteristics between different brand personalities and brand love?
4. How does brand experience sharing influence the eWOM on the internet.

This research contributes to the influence of brand love on brand electronic word-of-mouth from the perspective of brand personality. Furthermore, because brand personality is related to consumer personality categories, the function of different consumer personalities in brand personality and brand love is also involved. Furthermore, the research sets forth the relation between brand experience sharing and brand love, since the research setting is the knowledge payment industry based on the internet, which is relevant to the sharing and communication of knowledge.

## Literature Review

### Impact of Brand Personality on Brand Love

The definition of brand personality was initially put up by Gardner and Levy (1955), who believe that brand personality includes various perspectives of consumer personality, such as the consumer’s gender, age, and social status. These personalities derive directly from brand users or indirectly from users of other products. Sirgy (1982) defined that “Products, suppliers, and services are assumed to have an image determined not only by the object’s physical characteristics alone.” Keller (1993) pointed out that brand personality is shaped by producer and consumer together, instead of preserved by the product itself.

Consumers are willing to invest in similar brands, and their relationships (Keller, 1998). Brand personality is the soul of the brand and the unique competitiveness of the brand. The shaping of brand character evokes consumers’ emotions (Fournier, 1998) and helps consumers distinguish between products and brands and make differentiated choices (Farquhar, 1990).

In order to study more about cultural differences in perceiving brand personality, Aaker (1997) explored and examined dimensions of brand personality toward multiple international brands, thus dividing brand personality into five scales: sincerity, excitement, competence, sophistication, ruggedness.

Fournier and Mick (1999) brought up the concept of consumer satisfaction. However, it was not until Carroll and Ahuvia (2006) officially put up brand love. Carroll and Ahuvia (2006) also defined and measured brand love. Brand love refers to the strong affection from satisfied consumers toward one specific brand, and the affection includes enthusiasm, attachment, positive feelings, positive comments, and love. The definition of brand love explicitly proves that brand love is a positive consumer attitude to the brand and irrelevant to consumers who pose negative comments. Keh et al. (2007) explained that brand love is borrowed from the meaning of human love so that the relationship between consumers and brands is similar to human-to-human ties. Based on this principle, brand love is categorized into different dimensions, and most scholars approve of the categorization. This research employs Ortiz and Harrison (2011) definition of brand love, which believes that brand personality is critical for brand love and loyalty. At the same time, there is an emotional connection between consumer and brand. In addition, Rageh Ismail and Spinelli (2012) and Albert and Merunka (2013) showed that different dimensions of brand personality (sincerity, excitement, competence, sophistication, ruggedness) have a significant positive impact on brand love. Bairrada et al. (2020) research of Portuguese consumers of clothing brands concluded that brand personality significantly affects brand love, resisting negative information, and self-disclosure. Shetty and Fitzsimmons (2021) research of high-income groups in Dubai showed that brand personality is the key deciding element of brand love and brand loyalty. Since brand personality is classified into five dimensions (Aaker, 1997; Aaker et al., 2001), this study proposes the following hypotheses:

H1: Brand personality has a positive influence on brand love.

H1-1∼H1∼5: The (1-1) competence, (1-2) sincerity, (1-3) excitement, (1-4) sophistication, and (1-5) ruggedness components of brand personality each have a significant, unique relationship with brand love.

### The Influence of Brand Love on Electronic Word-of-Mouth

Buttle (1998) reckoned that word of mouth is also essential in the digital age. Electronic bulletins in business competitions present electronic word-of-mouth(eWOM). eWOM is traceable and serves as free advertisements on the internet because electronic word-of-mouth is a new form of informal dissemination of specific service or goods information beyond the geographical limitation. Consumers can exchange their opinions after purchasing. eWOM communication can be spread on a large scale, anonymously, and timely (Stephen et al., 2008).

Zhu and Zhang (2012) asserted that when college students purchase branded products, brand love will positively influence word-of-mouth communication; self-identification brand positively influences brand love and word-of-mouth communication. From the statistics of 400 clients and questionnaires collected by a silk brand community, Hathairat and Anon (2016) concluded that brand love in the brand community affects word-of-mouth communication both directly and indirectly. Suthasinee and Anon (2015) in KhonKaen also collected 400 surveys targeting the relationship between brand love and word-of-mouth communication in an airline company AirAsia. The result shows that brand loyalty also, directly and indirectly, influences word-of-mouth communication. Kiuru (2014) also proved that brand love positively impacts eWOM and traditional word-of-mouth. In conclusion, brand love would influence the degree of word-of-mouth communication. Therefore, the research develops the following hypothesis.

H2: Brand love has a positive influence on positive eWOM.

### Mediating Effect of Brand Love

Rageh Ismail and Spinelli (2012) showed that brand love has positive influence on positive word-of-mouth communication; Roy et al. (2013) and Unal and Aydın (2013) both showed that brand love positively influences willingness to pay a premium price. Albert and Merunka (2013) showed a positive impact on brand commitment, positive word-of-mouth communication, and the tendency to pay a premium for a brand. Wallace et al. (2014) similarly demonstrated a positive impact of brand love on positive word-of-mouth communication and brand acceptance. Lee and Kim (2018) demonstrated that the impact of consumer self-awareness on consumer behavior intention is influenced by the role of brand love as an intermediary. Bairrada et al. (2020) explores the mediation effect of brand love from a study of Portuguese consumers. Liu (2020) researched that brand personalities’ sophistication, competence, and ruggedness positively influence consumers’ willingness to WOM. The sophistication depends entirely on the brand’s love to influence the willingness of word of mouth. Brand love has a mediating effect on the relationship of brand personality competence, and ruggedness can positively influence word-of-mouth communication. In conclusion, brand love has a positive influence on word-of-mouth communication. Thus, H3 is derived as follows:

H3: Brand love mediates the relationship between brand personality and eWOM.

### Moderated the Mediation Effect of Brand Experience Sharing

Ross and Darke (2002) officially raised the concept of brand experience, and they believe that brand experience is a face-to-face communication aiming at attracting consumers emotionally and materially, while Brakus et al. (2009) deemed that brand experience is a subjective and interior consumer reaction, including brand design, brand identification, wrappings, communication, environment. Hardin and Higgins (1996) proposed the shared reality theory. The “shared” refers to both sides reaching a balance emotionally by interacting with each other to share the same understanding of some social prospects. The “reality” means people’s subjective views toward real objects instead of the actual object itself (Echterhoff et al., 2009). Brand experience sharing is to share the real opinion after purchasing or using the brand services and products (Yang et al., 2014). Considering the characteristics of brand experience, the author of the report defines brand experience as the action or willingness to share experience obtained by enjoying paid online-knowledge products or services. The following hypothesis is derived.

H4: Brand experience sharing moderated the mediation effect in the relationship between brand love and eWOM.

## Research Methods

### Research Design

**FIGURE 1:**
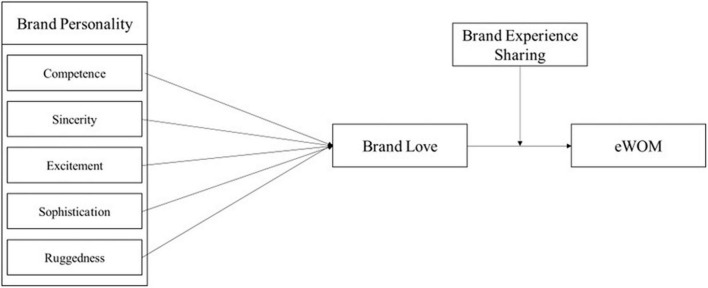
Research model.

This study examines the relationship between brand personality and eWOM in the context of iGT, mediating the role of brand love and moderation of brand experience sharing. The research model is presented in Figure 1.

### Data Collection and Measurement Design

The main research subject is the Chinese paid knowledge app: iGet, which boasts more than 28 million users. The statistics were collected by giving 540 online questionnaires from iGet’s official platform. Among 540 questionnaires, 539 questionnaires are answered, and 480 were valid, accounting for 89%.

The questionnaire includes brand personality, brand love rating, electronic word-of-mouth communication willingness, consumer personality, and brand experience sharing intention or behavior measurement section (Table 1). All items measured adopting a 7-point Likert scale ranging from point 1 = “strongly disagree” to point 7 = “strongly agree.” Firstly, this study measured the Big-5 brand personality (competence, sincerity, excitement, sophistication, ruggedness) proposed by Aaker (1997). Secondly, according to the brand love measurement developed by Carroll and Ahuvia (2006), brand love is uni-dimensional, including 10 indicators (eight positive and two negative items). Thirdly, electronic word-of-mouth is measured under the four items of the diffuse intention proposed by Carroll and Ahuvia (2006). Fourthly, the brand experience is measured under different behavioral rating scales of Eccles et al. (1983), Bock and Kim (2002), Battle and Wigfield (2003), Pempek et al. (2009), and Ross et al. (2009). At the end of the survey, demographic information was collected.

**TABLE 1:** Measurement scale.

Variable	Dimension	Sources
Brand personality	Sincerity	Aaker, 1997
	Excitement	
	Competence	
	Sophistication	
	Ruggedness	
Brand love		Carroll and Ahuvia, 2006
Positive eWOM		Carroll and Ahuvia, 2006
Brand experience		Eccles et al., 1983; Bock and Kim, 2002; Battle and Wigfield, 2003; Pempek et al., 2009; Ross et al., 2009

Valid samples are analyzed in terms of primary research takers, such as gender, age, time spent using the app, and other factors. Among all valid samples, 293 samples are male, accounting for 61% of the total; 144 samples aged from 20 to 29 years old, and another 144 samples aged from 30 to 39, each accounting for 30% of the total; 190 samples have earned bachelor’s degrees, accounting for 39.6% of the total; more than 22% work in the field of education; 60% have entered the work world for less than 3 years; 29.2% have 1001–2000 yuan disposable income per month. The majority, 38.1%, spend 3–5 h on iGet. Table 2 presents the demographic information of the sample.

**TABLE 2:** Demographic description of the sample (*N* = 480).

Attribute	Details	Number of people	Percentage
Gender	Male	293	61
	Female	187	39
Age	Under 18–20 years old	48	10
	20–29 years old	144	30
	30–39 years old	144	30
	40–49 years old	96	20
	50 years old and over	48	10
Educational attainment	Associate degree and below	52	10.8
	Bachelor’s degree	190	39.6
	Master’s degree	185	38.5
	Doctor’s degree and above	53	11
Marital status	Married	240	50
	Unmarried	192	40
	Divorced or widowed	48	10
Working sectors	IT	110	22.9
	Finance	38	7.9
	Service	91	19
	Education	134	27.9
	Retail	15	3.1
	Tourism	14	2.9
	Real-estate	19	4
	Others	59	12.3
Working age	Less than 1 year	144	30
	1–3 years	144	30
	3–5 years	96	20
	More than 5 years	48	10
	Zero	48	10
Disposable income per month	Less than 500 CNY	51	10.6
	501–1000 CNY	93	19.4
	1001–2000 CNY	140	29.2
	2001–3000 CNY	96	20
	More than 3000 CNY	100	20.8
Cumulative using hours per day	Less than 1 h	55	11.5
	1–3 h	139	29
	3–5 h	183	38.1
	More than 5 h	103	21.5

*Above all, iGet targets young adults with less working age, stronger cognition, and more spared time.*

## Empirical Results

### Reliability and Validity Analysis

Firstly, confirmatory factor analysis (CFA) was employed to test reliability and validity. The reliability analysis results for each scale show that all variables’ composite reliability (CR) is greater than 0.7, which values above 0.7 indicate good reliability (Bagozzi and Yi, 1988). The average variances extracted (AVE) of all constructs greater than 0.5 indicate good convergent validity of variables (Bagozzi and Yi, 1988). All variables’ Cronbach alpha was larger than 0.7 (Nunally and Bernstein, 1978). Finally, all constructs’ factor loading is higher than 0.5, which means good construct validity (Fornell and Larcker, 1981; Hair et al., 2013). Therefore, these results demonstrated reasonable reliabilities for these measured items.

Discriminant validity is shown when the following two criteria are met: (1) measurement items load more strongly on their assigned construct than on other constructs in a CFA, and (2) when the square root of the Average Variance Extracted (AVE) of each construct is larger than its correlations with other constructs (Gefen and Straub, 2005). As shown in Table 3, all measurement items are loaded much more strongly on their respective factors than on other constructs. Table 4 present the descriptive statistics.

**TABLE 3:** Reliability and validity analysis.

Construct	Dimensions	Items	Estimate	α	AVE	CR
Brand personality	Sincerity	I think iGet is utilitarian.	0.608	0.695	0.540	0.796
		I think iGet is healthy.	0.762			
		I think iGet has feelings.	0.665			
	Excitement	I think iGet is brilliant.	0.742	0.744	0.503	0.751
		I think iGet is unique.	0.705			
		I think iGet is young.	0.713			
	Competence	I think iGet is valuable.	0.750	0.732	0.583	0.735
		I think iGet is reliable.	0.758			
		I think iGet is progressive.	0.694			
	Sophistication	I think iGet is charming.	0.658	0.744	0.500	0.749
		I think iGet is successful.	0.725			
		I think iGet is fascinating.	0.636			
	Ruggedness	I think iGet is strong.	0.683	0.720	0.509	0.716
		I think iGet is outdoors.	0.683			
		I think iGet is masculine.	0.724			
Brand love		1. iGet is a good brand.	0.674	0.923	0.551	0.924
		I feel good about iGet.	0.763			
		iGet is respective.	0.786			
		I hold a neutral attitude to iGet. (R)	0.809			
		I am happy using iGet.	0.734			
		I love the brand: iGet.	0.670			
		iGet gives me pure happiness.	0.754			
		iGet fails to make myself feel special. (R)	0.738			
		I feel enthusiastic to iGet.	0.765			
		I am obsessed with iGet.	0.716			
Brand experience sharing		I feel proud of sharing iGet on social media.	0.711	0.817	0.533	0.820
		I think sharing iGet on moments can make myself be in the spotlight.	0.748			
		I make comments on others’ sharing of iGet.	0.667			
		I share my thoughts and experience of learning on iGet and hope to make more friends out of this way.	0.701			
eWOM		I recommend iGet to many friends.	0.715	0.800	0.502	0.801
		I share numerous information about iGet with others.	0.757			
		I try to communicate the advantages of iGet to others.	0.756			
		I provide positive word-of-mouth advertisements for iGet.	0.690			

**TABLE 4:** Descriptive statistics.

	Mean	S.D.	(1)	(2)	(3)	(4)	(5)	(6)	(7)	(8)
Competence (1)	5.637	0.964	1							
Sincerity (2)	5.233	1.107	0.649**	1						
Excitement (3)	5.710	0.990	0.800**	0.645**	1					
Sophistication (4)	5.463	1.029	0.782**	0.715**	0.802**	1				
Ruggedness (5)	5.528	1.004	0.831**	0.737**	0.783**	0.813**	1			
Brand experience sharing (6)	5.441	1.040	0.681**	0.735**	0.698**	0.696**	0.744**	1		
Brand love (7)	5.473	0.979	0.812**	0.791**	0.813**	0.847**	0.849**	0.820**	1	
eWOM (8)	5.409	0.988	0.664**	0.755**	0.687**	0.723**	0.753**	0.849**	0.827**	1

** p < 0.05.

### Structural Model and Hypothesis Testing

**FIGURE 2:**
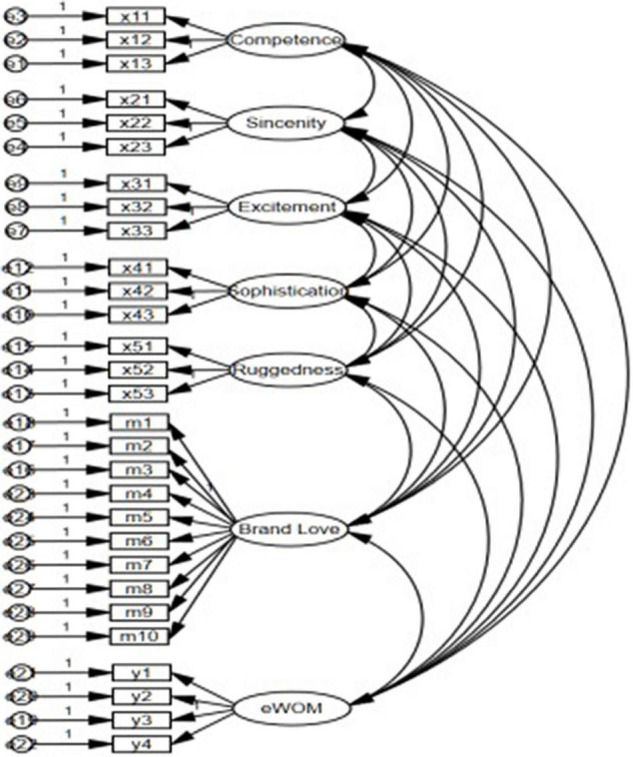
Measurement model.

**FIGURE 3:**
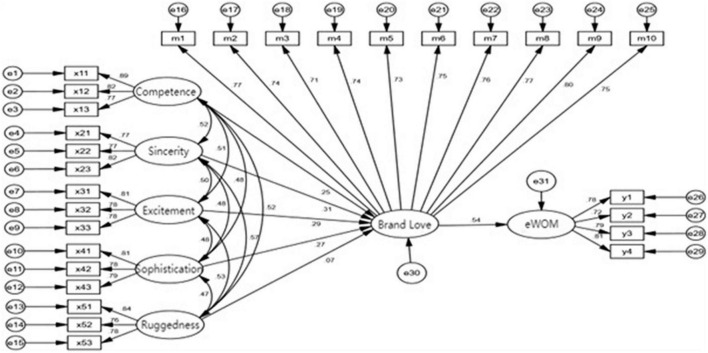
SEM model.

The initial structural equation model must be constructed before the empirical test. The initial model constructed in this research can be divided into a structural model and a measurement model, where the central task is to construct the structural model. It is necessary to determine the relationship among and role of each variable before constructing the initial model. This manuscript’s latent variables are competence, sincerity, excitement, sophistication, ruggedness, brand love, and electronic word-of-mouth. When the structural model has been constructed, a measurement model is needed to predetermine the relation between each latent variable and its corresponding observed variable(item). In this manuscript, the conceptual model is combined with several research hypothesis considerations in the initial model construction, and the measurement model shown in Figure 2 below is now constructed in order to lay the foundation for the empirical test. The calculations are conducted by AMOS 23.0, and Figure 3 is obtained.

In the following step, this study tested the goodness of fit of the constructed models. From Table 5, it can be seen that the value of CMIN/DF is 1.334, less than the criterion of 3. AGFI, GFI, NFI, TLI, IFI, and CFI are all above the criteria of 0.9. The value of RMR is 0.041, which is less than 0.08, and RMSEA is 0.026, which is less than 0.06. Each fit index meets the general research criteria, so it can be considered that this model has a good fit.

**TABLE 5:** SEM empirical results.

Hypothesis	Path	Estimate	S.E.	*P*-value	Result
H_1–1_	Competence → Brand love	0.246	0.028	***	Significant
H_1–2_	Sincerity → Brand love	0.311	0.033	***	Significant
H_1–3_	Excitement → Brand love	0.291	0.029	***	Significant
H_1–4_	Sophistication → Brand love	0.265	0.027	***	Significant
H_1–5_	Ruggedness → Brand love	0.073	0.027	*	Partially significant
H2	Brand love → eWOM	0.541	0.061	***	Significant

*** p < 0.001, * p < 0.100.

The analysis results reveal that competence has a significant poistive effect on brand love (*b* = 0.246, *p* < 0.05) supporting H_1–1_; sincerity has a significant positive impact on brand love (β = 0.311, *p* < 0.05) supporting H_1–2_; excitement has a significant positive impact on brand love (β = 0.291, *p* < 0.05) supporting H_1–3_; sophistication has a significant positive impact on brand love (β = 0.265, *p* < 0.05) supporting H_1–4_; ruggedness has partially significantly affect brand love (β = 0.073, *p* < 0.10) supporting H_1–5_. The path analysis results show that brand love significantly and positively affect eWOM (β = 0.541, *p* < 0.05), supporting H_2_.

### Mediation Analysis

This study the adopted Bootstrap method to test the mediation effect (Model 4; Hayes, 2017). The mediation analysis results (Table 6) show that brand love mediates the relationship between overall brand personality and eWOM (*b* = 0.574, 95% Boot CI = [0.209, 0.794], excluding 0). Ruggedness mediates the relationship between overall brand personality and eWOM (*b* = 0.106, 95% Boot CI = [0.019, 0.244], excluding 0). Sophistication mediates the relationship between overall brand personality and eWOM (*b* = 0.127, 95% Boot CI = [0.023, 0.241], excluding 0). Sincerity mediates the relationship between overall brand personality and eWOM (*b* = 0.095, 95% Boot CI = [0.012, 0.209], excluding 0). Competence mediates the relationship between overall brand personality and eWOM (*b* = 0.082, 95% Boot CI = [−0.022, 0.178]). The index of mediation shows that the brand love mediates the relationship between brand personality and eWOM supporting H_3_.

**TABLE 6:** Mediation analysis results.

Moderation	Indirect effect	*P*	95% Boot CI
Brand love → Brand personality → eWOM	0.574	0.000	0.209∼0.794
Brand love → Ruggedness → eWOM	0.106	0.000	0.019∼0.244
Brand love → Sophistication → eWOM	0.127	0.000	0.023∼0.241
Brand love → Excitement → eWOM	0.095	0.000	0.012∼0.209
Brand love → Sincerity → eWOM	0.129	0.000	0.023∼0.293
Brand love → Competence → eWOM	0.082	0.000	−0.022∼0.178

### Brand Experience Sharing Moderated Mediation

This study conducted multiple serial regression and spotlight analyses to test the moderated mediation effect. The Independent variable is brand love; brand experience sharing is moderator; the dependent variable is eWOM. Gender, age, educational attainment, marital status, working sectors, the working year, disposable income per month, and cumulative using hours per day served as control variables.

To test moderated mediation effect, this study employed Process macro Model 14 (Hayes, 2017). The index of moderated mediation shows that the brand experience sharing moderates the mediation role of the effect of brand love on eWOM supporting H_4_. In the high level of brand experience sharping condition(M-1SD), the brand experience sharing moderated the indirect effect of brand personality on eWOM through brand love (β = 0.285, 95% CI = [0.001. 0.557], excluding 0), but not in high brand experience sharing(M + 1SD) (β = 0.296, 95% CI = [−0.029. 0.597]) (Table 7). Therefore, brand experience sharing moderated the mediation of brand personality between brand personality and eWOM.

**TABLE 7:** Conditional indirect effect.

	Effect	Boot SE	Boot LLCI	Boot ULCI
Low level (−1SD)	0.285	0.141	0.001	0.557
Mean	0.291	0.149	−0.011	0.574
High level (+1SD)	0.296	0.161	−0.029	0.597

## Conclusion and Discussion

### Conclusion

According to the results, it can be concluded that ruggedness, competence, sincerity, excitement, and sophistication are brand personalities in line with what has been described as brand personalities of iGet. They also positively affect consumers’ brand love for the platform, while ruggedness, as a brand personality, has partially significant effect on the platform consumers. Ruggedness partially affects brand love because Aaker (1997) description of “ruggedness” is relatively masculine, not exactly what iGet presents. On the other hand, it may be that 40% of sample data is contributed by women, whose impacts cannot be neglected. Furthermore, the question items about brand personality used in this manuscript come from scholars’ tests of the western world and may not be perfectly suitable for Chinese consumers, considering that the internet is a special business environment. In this research, the Internet brand iGet has sincerity, competence, excitement, and sophistication as brand personalities that can generate consumers’ brand love, while ruggedness, as a brand personality, cannot.

In this research, brand love has a significant positive effect on electronic word-of-mouth. The conclusion of this research shares many commons with what Kiuru (2014) has put forward. She mentions that brand love positively affects electronic word-of-mouth, but nuances remain as this research is conducted in different industries and has different samples. Since iGet has been launched, live streaming, off-line speech, and mutual recommendation are its marketing strategies to improve consumer conversion rate. This word-of-mouth can subconsciously spread brand values straight to consumers’ hearts, so “word-of-mouth marketing” can be realized by those consumers who have strong satisfaction with the brand.

Based on this research, it can be found that brand experience sharing has a significant impact on the effect of brand love on electronic word-of-mouth. Brand experience is like face-to-face communication between a brand and consumers at the emotional and material level (Ross et al., 2009). Moreover, on the internet, a virtual environment, the brand experience is more about the impact of information on consumers’ senses and perceptions. In this sense, it is easier to share informationized brand experience. Pham and Huynh (2017) have studied the use of Facebook in Vietnam as a background and concluded that sharing information through SNS has a positive impact on WOM, while sharing iGet is basically through SNS like WeChat link. In this way, when users of iGet have both software (brand experience) and hardware (WeChat sharing), brand experience sharing will be a natural thing. For a brand frequently utilizing word-of-mouth marketing like iGet, brand experience sharing certainly significantly impacts the effect of brand love on electronic word-of-mouth.

### Implications

This research proves that brand variables like brand personality and brand love play important roles even in Internet knowledge payment brands. As long as it has distinctive personalities, an electronic brand will still make consumers feel a sense of deep connection. Because of the characteristics of this industry, the spread of brand word-of-mouth has changed from traditional methods into electronic ones, making the spread of electronic word-of-mouth convenient and far-reaching, and brand love on the internet can also affect electronic word-of-mouth. This research innovatively adds the moderation of brand experience sharing in brand love and electronic word-of-mouth. As a result, it is verified that among online knowledge payment brands, brand experience sharing can promote positive electronic word-of-mouth based on consumers’ brand love they have already had.

From the perspective of online users, this research demonstrates that brand personalities can generate different perceptions and emotions about the brand. In this sense, they will consistently share the brand’s word-of-mouth while disseminating brand information. This conclusion is undoubtedly a reminder to enterprises to upgrade their brands and establish their images. In today’s online world, where consumers’ attention is limited, brands that cannot combine “interesting” and “informative” may not be able to attract users’ attention, let alone generate emotional connections. If a business brand does not have word-of-mouth publicity from its “regular customers,” it would be hard to increase its market awareness quickly, and some of the marketing methods followed will not be developed.

### Limitations and Further Research

Although this research has yielded meaningful findings on the relationship between brand personality and brand word-of-mouth, it has the following limitations. Firstly, this research is conducted by targeting the Chinese online platform iGet alone and it is necessary to study consumers in other countries and various platforms in further research. Based on the characteristics of consumers in different counties and the features of platforms, there will be different relationships between brand personalities and brand word-of-mouth. Therefore, considering the situation mentioned above, it is necessary to use various platforms as objects in the following research, and valuable experience and brand trust can be added further to discuss the relationships between brand personalities and brand word-of-mouth. The electronic brand is a comparatively new discipline for research. With the in-depth development of the internet, more and more electronic brands and their electronic WOM will emerge. Brand marketing will become the foundation of enterprises in the future. We hope that the follow-up research will provide richer insights through various platforms and factors.

## Data Availability Statement

The raw data supporting the conclusions of this article will be made available by the authors, without undue reservation.

## Author Contributions

ML: formal analysis, writing—original draft preparation, and supervision. JY: visualization and project administration. Both authors contributed to the conceptualization, methodology, validation, data curation, writing—review and editing, and read and agreed to the published version of the manuscript.

## Conflict of Interest

The authors declare that the research was conducted in the absence of any commercial or financial relationships that could be construed as a potential conflict of interest.

## Publisher’s Note

All claims expressed in this article are solely those of the authors and do not necessarily represent those of their affiliated organizations, or those of the publisher, the editors and the reviewers. Any product that may be evaluated in this article, or claim that may be made by its manufacturer, is not guaranteed or endorsed by the publisher.

## References

[B1] Aaker J. L. (1997). Dimensions of Brand Personality. *J. Mark. Res.* 34 347–356. 10.1177/002224379703400304

[B2] Aaker J. L. Benet-Martinez V. Garolera J. (2001). Consumption Symbols as Carriers of Culture: a Study of Japanese and Spanish Brand Personality Constructs. *J. Personal. Soc. Psychol.* 81 492–508. 10.1037/0022-3514.81.3.492 11554649

[B3] Albert N. Merunka D. (2013). The Role of Brand Love in Consumer-Brand Relationships. *J. Cons. Mark.* 30 258–266. 10.1108/07363761311328928

[B4] Arnaldo C. Cristela B. Filipa P. (2019). Brand Communities’ Relational Outcomes, Through Brand Love. *J. Prod. Brand Manag.* 28 154–165.

[B5] Bagozzi R. P. Yi Y. (1988). On the Evaluation of Structural Equation Models. *J. Acad. Mark. Sci.* 16 74–94. 10.1007/BF02723327

[B6] Bairrada C. M. Coelho A. Lizanets V. (2020). The impact of brand personality on consumer behavior: the role of brand love. *J. Fashion Mark. Manage*. 23, 30–47. 10.1108/JFMM-07-2018-0091

[B7] Battle A. Wigfield A. (2003). College Women’S Value Orientations toward Family, Career, and Graduate School. *J. Vocat. Behav.* 62 56–75. 10.1016/S0001-8791(02)00037-4

[B8] Bock G. W. Kim Y. G. (2002). Breaking the Myths of Rewards: an Exploratory Study of Attitudes about Knowledge Sharing. *Inform. Manag. Res. J.* 15 14–21. 10.4018/irmj.2002040102

[B9] Brakus J. J. Schmitt B. H. Zarantonello L. (2009). Brand experience: what is it? How is it measured? Does it affect loyalty? *J. Mark.* 73 52–68. 10.1509/jmkg.73.3.52 11670861

[B10] Buttle F. A. (1998). Word of Mouth: understanding and Managing Referral Marketing. *J. Strat. Mark.* 6 241–254. 10.1080/096525498346658

[B11] Carroll B. A. Ahuvia A. C. (2006). Some Antecedents and Outcomes of Brand Love. *Mark. Lett.* 17 79–89. 10.1007/s11002-006-4219-2

[B12] Eccles J. Adler T. F. Futterman R. Goff S. B. Kaczala C. M. Meece J. (1983). “Expectancies, Values and Academic Behaviors,” in *Achievement and Achievement Motives*, ed. Spence J. T. (San Francisco, CA: W. H. Freeman), 75–146.

[B13] Echterhoff G. Higgins E. T. Levine J. M. (2009). Shared reality: experiencing commonality with others’ inner states about the world. *Perspect. Psychol. Sci.* 4 496–521. 10.1111/j.1745-6924.2009.01161.x 26162223

[B14] Farquhar P. H. (1990). Managing Brand Equity. *J. Adv. Res.* 30 7–12.

[B15] Fornell C. Larcker D. F. (1981). Structural Equation Models with Unobservable Variables and Measurement Error: algebra and Statistics. *J. Mark. Res.*. XVIII, 382–388. 10.1177/002224378101800313

[B16] Fournier S. (1998). Consumers and Their Brands: developing Relationship Theory in Consumer Research. *J. Cons. Res.* 24 343–373. 10.1086/209515

[B17] Fournier S. Mick D. G. (1999). Rediscovering Satisfaction. *J. Mark.* 63 5–23. 10.1177/002224299906300403

[B18] Gardner B. B. Levy S. J. (1955). The Product and the Brand. *Harv. Bus. Rev.* 33 33–39.

[B19] Gefen D. Straub D. (2005). A Practical Guide to Factorial Validity Using PLS-Graph: tutorial and Annotated Example. *Comm. Assoc. Inform. Syst.* 16:5. 10.17705/1CAIS.01605

[B20] Hair J. F. Ringle C. M. Sarstedt M. (2013). Partial Least Squares Structural Equation Modeling: rigorous Applications, Better Results and Higher Acceptance. *Long Range Plan.* 46 1–12. 10.1016/j.lrp.2013.01.001

[B21] Hardin C. D. Higgins E. T. (1996). *Shared reality: How Social Verification Makes the Subjective Objective*, Vol. 99. New York, NY: Guilford Press, 452–466.

[B22] Hathairat N. Anon K. (2016). Brand Community, Brand love, and Word of Mouth: a Case of Surin Silk Brand. *Conf. Internat. J. Art Sci.* 2016 261–268.

[B23] Hayes A. F. (2017). *Introduction to Mediation, Moderation, and Conditional Process Analysis: A Regression-Based Approach.* New York, NY: Guilford Publications.

[B24] Keh H. T. Pang J. Peng S. (2007). “Understanding and Measuring Brand Love,” in *Society for Consumer Psychology Conference Proceedings*, (Santa Monica), 84–88.

[B25] Keller K. L. (1993). Conceptualizing, Measuring, and Managing Customer Based Brand Equity. *J. Mark.* 57 1–22. 10.1177/002224299305700101

[B26] Keller K. L. (1998). *Strategic Brand Management.* New Jersey: Prentice Hall. 62 48–57.

[B27] Kiuru K. (2014). The Relationship Between Brand Love and Positive Word Of Mouth. *J. Mark.* 45 15–37.

[B28] Lee J. H. Kim H. K. (2018). The Effects of Consumer’s Self-Awareness on Behavior Intention: focusing on the Moderating Effect of Brand Personality and Mediating Effect of Brand Love. *J. Bus. Res.* 33 107–133.

[B29] Li J. Wang Q. Chen Z. (2012). Review of brand Theory research. *Enterprise Reform Manag.* 20 12–13. 10.1057/bm.2012.3

[B30] Liu M. (2020). The Role Mechanism of Brand Personality on Word Of Mouth Communication: the Formation and Expression of Brand Attachment. *Enterp. Econ.* 2020 67–75.

[B31] Mehrabian A. Russell J. A. (1974). *An Approach to Environmental Psychology.* Cambridge, MA: MIT Press.

[B32] Nunally J. C. Bernstein I. H. (1978). *Psychometric Theory.* New York, NY: McGraw-Hill.

[B33] Ortiz M. H. Harrison M. P. (2011). Crazy Little Thing Called Love: a Consumer-Retailer Relationship. *J. Mark. Dev. Compet.* 5 68–80.

[B34] Pempek T. A. Yermolayeva Y. A. Calvert S. L. (2009). College Students’ Social Networking Experiences on Facebook. *J. Appl. Dev. Psychol.* 30 227–238. 10.1016/j.appdev.2008.12.010

[B35] Pham Q. T. Huynh V. K. (2017). “The Impacts of Using SNSs on e-WOM and Knowledge Sharing Through Social Capital: An Empirical Study in Vietnam,” in *Computational Science and Its Applications – ICCSA 2017. ICCSA 2017. Lecture Notes in Computer Science*, Vol. 10409 (Cham: Springer). 10.1007/978-3-319-62407-5_8

[B36] Rageh Ismail A. Spinelli G. (2012). Effects of Brand Love, Personality, and Image on Word Of Mouth: the Case of Fashion Brands Among Young Consumers. *J. Fash. Mark. Manag.* 16 386–398. 10.1108/13612021211265791

[B37] Ross C. Orr E. S. Sisic M. Arseneault J. M. Simmering M. G. Orr R. R. (2009). Personality and Motivations Associated with Facebook Use. *Comp. Hum. Behav.* 25 578–586. 10.1016/j.chb.2008.12.024

[B38] Ross J. Darke S. (2002). The Nature of Benzodiazepine Dependence among Heroin Users in Sydney, Australia. *Addiction* 95 1785–1793. 10.1046/j.1360-0443.2000.951217858.x 11177494

[B39] Roy S. K. Eshghi A. Sarkar A. (2013). Antecedents and Consequences of Brand Love. *J. Brand Manag.* 20 325–332. 10.1057/bm.2012.24

[B40] Shetty K. Fitzsimmons J. R. (2021). The Effect of Brand Personality Congruence, Brand Attachment and Brand Love on Loyalty among Henry’s in The Luxury Branding Sector. *J. Fash. Mark. Manag.* 53 458–465.

[B41] Sirgy M. J. (1982). Self-concept in Consumer behavior: a Critical Review. *J. Cons. Res.* 9 287–300. 10.1086/208924

[B42] Stephen W. Litvin R. E. G. Bing P. (2008). Electronic Word-of-Mouth in Hospitality and Tourism Management. *Tour. Manag.* 39 458–468. 10.1016/j.tourman.2007.05.011

[B43] Suthasinee N. Anon K. (2015). “Brand Love, Brand Loyalty and Word of Mouth: A Case of Airasia,” in *International Journal of Arts & Sciences*’ (IJAS) *International Conference for Business and Economics*, (Rome), 263–268.

[B44] Unal S. Aydın H. (2013). An Investigation on The Evaluation of the Factors Affecting Brand Love. *Procedia-Soc. Behav. Sci.* 92 76–85. 10.1016/j.sbspro.2013.08.640

[B45] Wallace E. Buil I. de Chernatony L. (2014). Consumer Engagement with Self-Expressive Brands: brand Love and WOM Outcomes. *J. Prod. Brand Manag.* 23 33–42. 10.1108/JPBM-06-2013-0326

[B46] Wang X. Guo J. Zhou C. (2018). Research on Knowledge Paid Products Based on Mobile Internet. *China Educ. Inform.* 24 19–25. 10.1155/2014/647370 24683349 PMC3934083

[B47] Yang D. Zhao P. Zhang M. (2014). Can Sharing the Brand Experience with Others Strengthen the Sharer’S Original Brand Attitude The Impact of -Experience Sharing on the Brand Attitude. *J. Mark. Sci.* 10 82–98.

[B48] Zhu H. Zhang X. (2012). Study on Prefactor and Consequence Variables of Brand Love. *Tax. Econ.* 33 29–35.

